# *In Vivo* Demonstration of Addressable Microstimulators Powered by Rectification of Epidermically Applied Currents for Miniaturized Neuroprostheses

**DOI:** 10.1371/journal.pone.0131666

**Published:** 2015-07-06

**Authors:** Laura Becerra-Fajardo, Antoni Ivorra

**Affiliations:** Department of Information and Communication Technologies, Universitat Pompeu Fabra, Barcelona, Spain; Duke University, UNITED STATES

## Abstract

Electrical stimulation is used in order to restore nerve mediated functions in patients with neurological disorders, but its applicability is constrained by the invasiveness of the systems required to perform it. As an alternative to implantable systems consisting of central stimulation units wired to the stimulation electrodes, networks of wireless microstimulators have been devised for fine movement restoration. Miniaturization of these microstimulators is currently hampered by the available methods for powering them. Previously, we have proposed and demonstrated a heterodox electrical stimulation method based on electronic rectification of high frequency current bursts. These bursts can be delivered through textile electrodes on the skin. This approach has the potential to result in an unprecedented level of miniaturization as no bulky parts such as coils or batteries are included in the implant. We envision microstimulators designs based on application-specific integrated circuits (ASICs) that will be flexible, thread-like (diameters < 0.5 mm) and not only with controlled stimulation capabilities but also with sensing capabilities for artificial proprioception. We *in vivo* demonstrate that neuroprostheses composed of addressable microstimulators based on this electrical stimulation method are feasible and can perform controlled charge-balanced electrical stimulation of muscles. We developed miniature external circuit prototypes connected to two bipolar probes that were percutaneously implanted in agonist and antagonist muscles of the hindlimb of an anesthetized rabbit. The electronic implant architecture was able to decode commands that were amplitude modulated on the high frequency (1 MHz) auxiliary current bursts. The devices were capable of independently stimulating the target tissues, accomplishing controlled dorsiflexion and plantarflexion joint movements. In addition, we numerically show that the high frequency current bursts comply with safety standards both in terms of tissue heating and unwanted electro-stimulation. We demonstrate that addressable microstimulators powered by rectification of epidermically applied currents are feasible.

## Introduction

The accumulated knowledge on the physiological principles behind sensory and motor neurons has opened the path to design medical devices that purposely apply electrical currents to elicit neural activity. In this way, the function of an impaired nervous system can be replaced or improved [1]. This therapeutic approach, known as Functional Electrical Stimulation (FES), has a tremendous potential to offer solutions for patients with neurological disorders caused by ailments such as spinal cord injury and stroke, among others. It has been estimated that every year between 250,000 and 500,000 people suffer a spinal cord injury [2]. Additionally, every year 795,000 people suffer a stroke in the United States, which makes it the leading cause of disability in the country [3]. Nowadays, some FES solutions are available for these patients [4]. They include systems for bladder and bowel control, as the Finetech-Brindley sacral root stimulator [5,6]; superficial FES devices for foot-drop, as the NESS L300 from Bioness [7]; and neuroprostheses for standing and walking, which are still under research [8].

FES systems can be coarsely classified into three categories according to the location of the pulse generator (i.e. the electronic system generating voltage or current pulses for stimulation) and the electrodes: surface, percutaneous and implanted. In surface systems, the pulse generator is external and the electrodes are attached to the patient’s skin. This configuration offers the least level of invasiveness but has three major disadvantages: it lacks spatial selectivity, it causes activation of subcutaneous pain receptors and it is difficult to don and doff [9]. Spatial selectivity is obtained in percutaneous systems. In this configuration, electrodes anchored to excitable tissues are connected to the external generator using wires that pierce the skin. Nevertheless, percutaneous systems are almost exclusively used for clinical diagnosis and research purposes because they cause infections and imply cosmetic issues. Nowadays, when spatial selectivity is required, fully implantable systems are largely preferred over the two other configurations. In this case, both the pulse generator and the electrodes are implanted within the patient’s body, avoiding risk of infections due to skin piercing, and affording spatial selectivity.

Electrical stimulation systems with implantable configuration are the least disadvantageous option in many clinically relevant scenarios. Implantation of most of these systems requires complex surgeries, hampering their use in pathologies for which less invasive treatment alternatives exist; even if these alternatives are suboptimal. Further research is needed in order to enhance implantable FES technologies, causing less injury to the body (e.g. deployment using percutaneous injection) and improving muscle recruitment.

Implantable systems consisting of central stimulation units wired to remote electrodes are not adequate for applications in which a large number of targets must be individually stimulated over large and mobile body parts, thus hindering solutions for patients suffering from paralysis [10]. As an alternative, in the 90s, it was proposed the development of addressable single-channel wireless microstimulators to be deployed by injection into the muscles to be stimulated [11]. These microstimulators were meant to form a dense network to be activated by an external automated controller so that fine motion restoration was achievable.

Until now, the microstimulators developed according to the above distributed network strategy have been either inductively powered [12] or battery powered [13]. Both methods result in devices which are considerably bulky (diameters ≥ 2 mm, lengths ≥ 16 mm) and stiff. These two features imply that the implants are quite invasive and this impedes dense deployment which in turn hampers selectivity.

The aim of this paper is to *in vivo* demonstrate the feasibility of a neuroprosthetic system composed of addressable microstimulators that rectify epidermically applied high frequency (HF) current bursts. The microstimulators can be implanted by percutaneous injection. This heterodox method for electrical stimulation–which is detailed in the next section–has the potential to enable unprecedented levels of miniaturization.

For the *in vivo* demonstration, we have developed miniature external prototypes connected to a bipolar flexible probe which can be percutaneously implanted at the target of interest for experimentation. The prototypes are able to decode commands that are amplitude modulated on the HF (1 MHz) auxiliary current bursts; similarly to previous studies in which intrabody communications are achieved by conductive coupling [14,15]. The prototypes were tested in an anesthetized rabbit, accomplishing controlled dorsiflexion and plantarflexion joint movements.

In addition, a short numerical analysis is included which predicts that the HF current bursts comply with safety standards both in terms of tissue heating and unwanted electro-stimulation.

## Electrical Stimulation Based on Electronic Rectification of Epidermically Applied Currents

**Fig 1:**
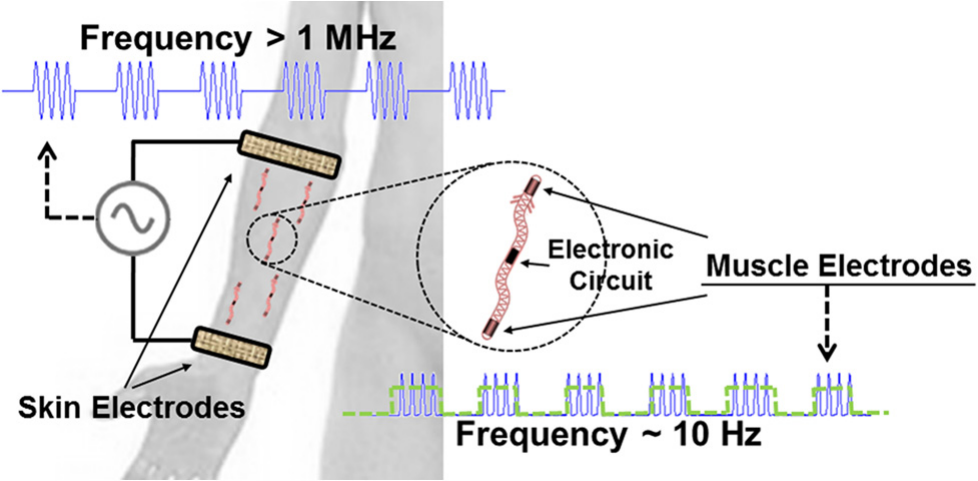
Schematic representation of the electrical stimulation method based on electronic rectification of epidermically applied currents. The external system supplies inert high frequency current bursts (> 1 MHz) to the tissues using skin electrodes. The implants pick-up the high frequency current bursts using their two peripheral electrodes (“muscle electrodes”), and rectify these currents, generating locally low frequency currents capable of stimulating excitable tissues.

In “Remote Electrical Stimulation by Means of Implanted Rectifiers” [16] we proposed a heterodox method for developing implantable microstimulators that avoids the use of inductive coupling or electrochemical batteries. Innocuous HF current bursts (> 1 MHz) are conductively supplied by skin electrodes across the tissues where the implants are located (Fig 1). The implants act as rectifiers of these HF current bursts, generating locally low frequency (LF) currents capable of stimulating excitable tissues.

The proposed method has the potential to enable unprecedented levels of miniaturization, accomplishing much thinner implants than those powered by inductive coupling or batteries. This is possible because only two peripheral electrodes (hereinafter “muscle electrodes”) are needed both for picking-up the HF current and for performing electrical stimulation. In addition, a tiny hybrid microcircuit, or a single integrated circuit, can integrate all the necessary electronic components. No bulky parts such as coils or batteries are required.

The proposed method requires a minimum voltage drop between the two muscle electrodes to power up the microstimulator and to generate the stimulating LF currents. This implies a minimum separation distance (in the order of a very few centimeters) between the two muscle electrodes to avoid the need of excessively large HF currents which would significantly heat the tissues. To ensure this, we conceive the implants as elongated bodies consisting of flexible and stretchable materials. Their mechanical properties match those of the tissue in which they are implanted. Because of such characteristic and their functionality, we named these implants “Electronic Axons” (eAXONs).

The first demonstrations of the electrical stimulation method were performed using an implant consisting of a single diode [16,17]. Such simple configuration is not adequate for clinical purposes because direct currents (DC) are generated through the implant and those can electrochemically damage both the tissues and the electrodes. Hence we recently developed and demonstrated implants capable of overcoming that drawback whose electronics consists of a diode, a capacitor and a resistor [18]. These implants (1 × 30 mm), which are flexible and can be percutaneously implanted by means of a thin catheter, could be used clinically in cases in which a single target needs to be stimulated (e.g. peripheral nerve stimulation for treatment of chronic pain). In particular, we deem that these implants, thanks to their miniature size, could be very valuable for the development of the so-called electroceuticals [19].

However, single target stimulation is not sufficient for movement restoration in paralysis patients. We envision neuroprosthetic systems in which eAXONs will be deployed forming a dense network of microstimulators that will be individually controlled by an autonomous external unit. This unit will deliver the required innocuous HF current bursts and will individually command each microstimulator. The implantable devices will perform complex stimulation patterns in a number of muscles or in segments of a muscle, as those required for fine movement restoration in patients suffering from paralysis [20]. A rough prototype of an addressable neuroprosthetic system based on this electrical stimulation concept was tested previously in an invertebrate model [21]. Yet this model only vaguely resembled a human body for conductive coupling evaluation (e.g. differences in skin impedance, tissue conductivity, and geometry), which could have a major impact in the proposed electrical stimulation method. In addition, the invertebrate model could present lower current thresholds for electrical stimulation. As a consequence, the system’s feasibility in a human could be questioned. The *in vivo* demonstration presented here uses a vertebrate model that resembles more closely a human model, and so, resolves these drawbacks. It effectively shows the feasibility of the addressable neuroprosthetic system based on the rectification of epidermically applied currents.

## Architecture of the Prototypes

**Fig 2:**
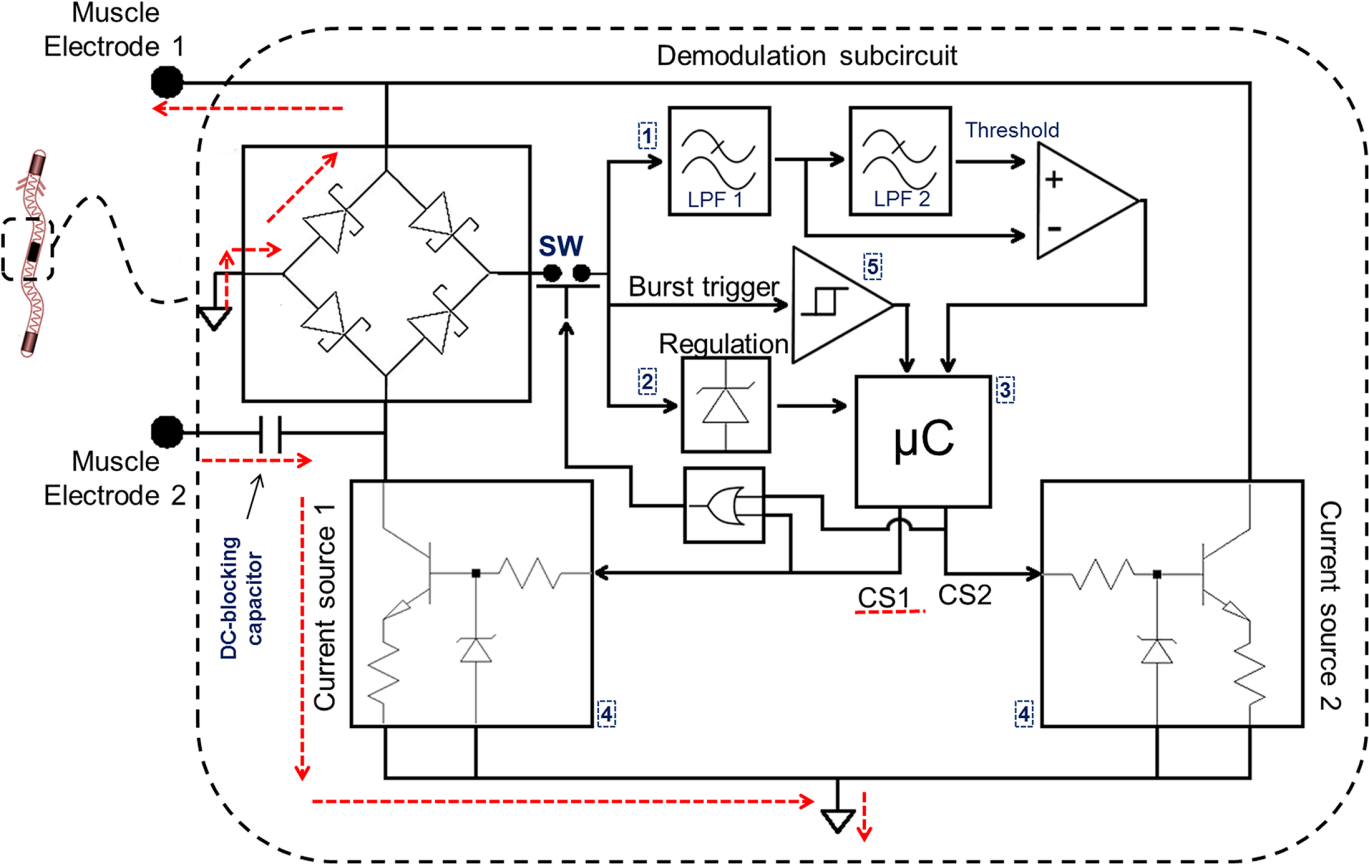
Architecture of the developed circuit prototypes for the microstimulators. The dashed red line represents the flow of stimulating (half-wave) rectified current when control signal 1 (CS1) activates the current source 1. If no current source is active, the switch (SW) closes and the alternating current (AC) picked-up by the muscle electrodes flows through the regulation subcircuit to power up the rest of the electronics. A demodulation subcircuit is used to extract information from the HF bursts, and a burst trigger is used to wake up the control unit when it is asleep in-between “Stimulation bursts”.

The architecture of the developed circuit prototypes for the implants is depicted in Fig 2. It consists of five main blocks: 1- a demodulator for the amplitude modulation (AM) communication system, 2- a power supply unit, 3- a digital control system (microcontroller), 4- two current sources able to generate biphasic currents and 5- a Schmitt trigger for waking up the digital control unit when a HF burst is detected.

The circuit prototypes were implemented using off-the-shelf components mounted on a pair of stacked rigid printed circuit board (PCB) breadboards (40 × 40 mm).

### Communications scheme

Amplitude-shift keying (ASK) is used to send data modulated on the HF (1 MHz) auxiliary current. In particular, Manchester coding was selected in order to guarantee a constant average amplitude (as required by the ASK demodulator described below) and for self-clocking.

**Fig 3:**
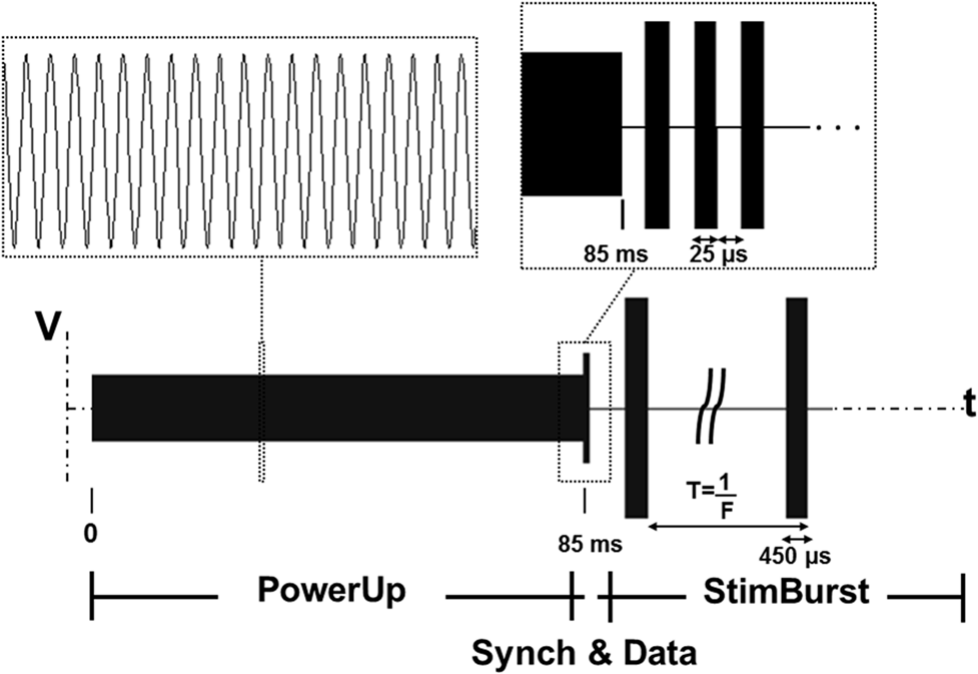
Representation of the ASK modulated voltage signal employed both for communications and for powering the circuit prototypes. It consists of three active stages: A) Power Up stage; B) Synchronization and Data stage, in which a specific circuit prototype is addressed and thereby activated; and C) Stimulation bursts stage, in which, for each burst, the activated circuit prototype delivers to tissues a biphasic symmetrical pulse of 200 + 200 μs with an interphase dwell of 30 μs.

The modulated 1 MHz signals consist of three distinguishable active stages (Fig 3) of specific relative amplitudes in order to minimize tissue heating (this is explained in detail in the Compliance of the Auxiliary Current with Safety Standards section). First, an 85 ms low amplitude unmodulated signal is used for the “Power up” stage. That is, for guaranteeing power up and stabilization of the whole circuitry and, in particular, of the microcontroller. Then, the “Power up” stage is followed by a 600 μs “Synch&Data” stage in which the control unit synchronizes and reads the information sent on the HF current. This stage consists of a sequence of three rising-edge transitions for synchronizing the decoder to the modulated signal and a 9 bit data stream (8 address bits and 1 parity bit). Bits are received at a baud rate of 20 kBd. After the “Synch&Data” stage, an 800 μs zero-amplitude slot is included for processing purposes. Processing tasks comprise decoding the information sent in the “Synch&Data” stage, the parity bit check, and comparing the decoded address with the programmed address in order to activate it for stimulation. At last, an unmodulated signal of maximum amplitude is used for the “Stimulation burst” stage. It is during these “Stimulation bursts” when LF currents (i.e. half-wave rectified alternating current (AC)) flow through the circuit and nerve stimulation is performed. The duration of these bursts (450 μs) is fixed in this study. The first 20 μs are employed for preprocessing purposes (control unit wake-up and power supply unit stabilization). Then, for 200 μs, rectified current flows from muscle electrode 1 to muscle electrode 2 (cathodic current according to our definition of the electrodes) and, after a brief slot of 30 μs in which no rectified current flows through the circuit, rectified current flows from electrode 2 to electrode 1 for 200 μs (anodic current). Therefore, a biphasic symmetric pulse of 200 + 200 μs is applied to tissues with an interphase dwell of 30 μs. This interphase dwell is a short time delay typically used in electrical stimulation between the cathodic and anodic pulse that allows the propagation of the action potential away from the stimulation site before the injected charge is recovered by the electrode [22]. The number of “Stimulation bursts” and their frequency F are variable and are determined by the user interface that sets the modulated signal (later explained).

It is only necessary to perform the “Power up” stage when initialization of the circuits is required. This is possible because the circuit enters a low power consumption mode (sleep mode) in-between “Stimulation bursts” and power is maintained by an internal capacitor. Therefore, once initial power up has been performed at the beginning of a stimulation session, no further “Power up” stages are required provided that “Stimulation bursts” are continuously delivered at a sufficient frequency (F > 20 Hz). Similarly, the “Synch&Data” stage only needs to be performed each time a different circuit must be activated for stimulation. That is, once a specific circuit prototype has been activated (i.e. selected by means of the address contained in the “Synch&Data” stage) no further “Synch&Data” stages are required until another circuit must be activated or initialization of all circuits (“Power up” stage) is required. This strategy is crucial to minimize the amount of effective current flowing through living tissues, therefore minimizing Joule heating and meeting safety standards. This is explained in depth in the Compliance of the Auxiliary Current with Safety Standards section.

The demodulator circuitry consists of two RC low-pass filters (series combination of a resistor and a capacitor) and a comparator that process the full-wave rectified signal from the electrodes. Low-pass filter 2 (cutoff frequency f_C2_ = 66.3 Hz) sets a threshold (amplitude average) that is compared to the signal filtered by the first low-pass filter (f_C1_ = 49.8 kHz > f_C2_). The output of the comparator (single-supply amplifier AD8605 by Analog Devices, Inc.) is then digitally processed in the microcontroller for Manchester decoding.

### Power supply

A full-bridge rectifier implemented with Schottky diodes (MCL103B-TR by Vishay Intertechnology, Inc.) provides full-wave rectification of the AC voltage picked up by the circuit’s electrodes. It is followed by a simple DC voltage regulation subcircuit consisting on a smoothing capacitor, a resistor and a zener diode. In-between “Stimulation bursts” the microcontroller, in sleep mode, is powered using a 47 μF capacitor.

During the stimulation stage, the regulation subcircuit is disconnected from the full-bridge rectifier using a NPN transistor switch (SW in Fig 2) in order to ensure that the generated LF current (half-wave rectified HF current) flows through the tissues rather than into the circuit.

### Digital control system

Aiming future miniaturization for implantation, a control system based on one of the smallest programmable devices was pursued. It was selected what nowadays appears to be the smallest commercially available programmable device: the ATtiny20 (Atmel Corp.). This integrated circuit is an 8 bit reduced instruction set computing (RISC) low-power microcontroller. One of its packaging options measures only 1.555 × 1.403 mm. It includes 10 input/output lines and software selectable power saving modes.

As depicted in Fig 2, two digital output lines of the microcontroller are used to switch on and off the two current sources and serve as inputs for an OR gate (SN74LVC1G32 by Texas Instruments, Inc.) that controls the power supply switch. A digital input is used as an external interrupt trigger in order to wake up the microcontroller when a new burst arrives. Another input is used for ASK demodulation.

The microcontroller of each prototype is programmed with a specific address of 8 bits. That is, only when the address received in the “Synch&Data” stream coincides with the programmed address, the prototype becomes active and stimulation is initiated.

### Current sources

Delivery of current pulses in order to perform electrical stimulation may elicit electrochemical damage both to the electrodes and to the tissue [23]. To avoid this, most implantable stimulators use magnitude-limited biphasic waveforms that perform zero net charge injection (i.e. the net DC component of the delivered current is zero). This has also been implemented here: the developed circuit prototypes include two independent current sources that generate complementary cathodic and anodic pulses (i.e. biphasic symmetric waveform).

Each current source consists of a zener diode that fixes a voltage in the base of a NPN transistor (Fig 2). This in turn fixes a voltage in the emitter of the transistor, defining a current flowing from the emitter to the resistor and ground. Then, rather than acting as a true DC current source, the circuit acts as a peak current limiter for the half-rectified HF current.

Since components tolerances, and in particular NPN transistors beta differences, can degrade matching between the two current sources, some degree of unbalanced charge injection could appear. Because of this, a dc-blocking capacitor (10 μF) was included for performing passive charge-balance of the unbalanced currents caused by current sources mismatching. The cathodic phase of the biphasic waveform charges the capacitor in one direction. When the anodic phase is enabled, it forces the dc-blocking capacitor’s discharge. If the injected charge of the first phase does not match the injected charge of the second phase, the remaining charge in the capacitor discharges passively through the implant and the tissues in-between stimulating bursts, compensating the injected misbalanced charge.

### Wake up signal

To minimize the amount of average HF current that flows through tissues, the microcontroller is programmed so that it enters a power-down sleep mode in-between stimulating bursts. For waking-up the device, the circuit includes a custom Schmitt Trigger based on a low power comparator (NCX2200 by NXP Semiconductors) that triggers an interrupt whenever a “Stimulation burst” is performed.

## Compliance of the Auxiliary Current with Safety Standards

A 1 MHz current may induce tissue heating and unwanted electro-stimulation. This is recognized by electrical safety standards which define maximum thresholds for preventing these potentially harmful effects [24,25]. Joule heating is limited in standards by specifying a maximum threshold for the so-called Specific Absorption Rate (SAR) whereas unwanted electro-stimulation is generally limited by specifying a maximum threshold for the electric field. For a hypothetical forearm neuroprosthetic system, we previously numerically demonstrated that an eAXON system may comply with electrical safety standards [26]. However, that analysis was performed under the assumption that power-up and communications slots were negligible in comparison to stimulation slots and that may not be the case with the prototypes developed here. Therefore, an *ad hoc* analysis is provided here to ensure that the ASK modulated HF current employed in this study does not cause excessive tissue heating nor unwanted electro-stimulation according to standards. In particular, the Institute of Electrical and Electronics Engineers (IEEE) safety standard was selected for reference [24].

The SAR in a tissue volume is defined as:

SAR=σ|Erms|2ρWkg (1)

Where *σ* is the tissue electrical conductivity (S/m), |**E**_rms_| is the applied electric field rms magnitude (V_rms_/m) and *ρ* is the tissue mass density (kg/m^3^).

As explained in Fig 3, the external system delivers an amplitude modulated signal with three different stages and a short processing time with different magnitudes and durations. Therefore, the SAR averaged over 1 second is:

$${\text{SAR}}_{1\;s}=\frac{\sigma }{\rho }\left(\int_{0}^{N\times 85\;ms}\;{\left|E_{\text{rms}}^{Power\;up}\right|}^{2}\;dt\;+\;\int_{0}^{n\times 0.6\;ms}{\left|E_{\text{rms}}^{Synch\&Data}\right|}^{2}\;dt+\int_{0}^{n\times 0.8\;ms}{\left|E_{\text{rms}}^{Processing\;Time}\right|}^{2}\;dt+\;\int_{0}^{1000-\left(N\times 85+n\times 1.4\right)ms}{\left|E_{\text{rms}}^{Stim}\right|}^{2}\;dt\right)$$ (2)

where *N* is the rate of initializations per second and *n* is the rate of activations per second (*n* ≥ *N*). Each term of the equation has the following values:

$$\int_{0}^{N\times 85\;ms}\;{\left|E_{\text{rms}}^{Power\;up}\right|}^{2}\;dt=N\times 0.085{\left|\frac{k_{1}E_{peak}}{\sqrt{2}}\right|}^{2}$$ (3)

$$\int_{0}^{n\times 0.6\;ms}\;{\left|E_{\text{rms}}^{Synch\&Data}\right|}^{2}\;dt=n\times 0.0006{\left|\frac{k_{2}E_{peak}}{\sqrt{2}}\right|}^{2}0.5$$ (4)

$$\int_{0}^{n\times 0.8\;ms}\;{\left|E_{\text{rms}}^{Processing\;Time}\right|}^{2}\;dt=0$$ (5)

$$\int_{0}^{1000-(N\times 85+n\times 1.4)\;ms}\;{\left|E_{\text{rms}}^{Stim}\right|}^{2}\;dt=\left(1-N\times 0.085-n\times 0.0014\right){\left|\frac{E_{peak}}{\sqrt{2}}\right|}^{2}D$$ (6)

where *k*_1_ is the relative amplitude of the applied electric field during the “Power up” stage (with respect to the amplitude of applied field, |**E**_*peak*_|, during stimulation), *k*_2_ is the relative amplitude during the “Synch&Data” stage and *D* is the duty cycle of the “Stimulation bursts” (*D* = *F* × 450 *μs*). Note that the 0.5 factor in Eq (4) corresponds to the duty cycle of the signal during the “Synch&Data” stage.

Performing *in vitro* experiments with saline phantoms, we found that, in order to generate stimulation pulses with an amplitude of about 2 mA, the amplitude of the HF voltage across the circuit electrodes must be 18 V. For an inter-electrode separation distance of 3 cm (i.e. distance between muscle electrodes), this implies that |**E**_*peak*_| must be 6 V/cm. On the other hand, the circuit prototypes are capable of proper initialization when the HF voltage across electrodes is just 10 V. Taking this into account we selected a value of 0.65 for *k*_1_. For *k*_2_ we selected a slightly larger value, 0.75. It compensates the discharge of the capacitors during the low levels of the “Synch&Data” stage and the processing slot.

Taking into consideration the above equations and parameters and assuming that the muscle conductivity at 1 MHz is 0.5 S/m [27] and its density is 1000 kg/m^3^ [28], the expected average SAR is given in Table 1 for different values of *N*, *n* and F.

**Table 1:** Expected Average SAR.

*N* (s^-1^)	*n* (s^-1^)	F (Hz)	SAR (W/kg)
1	1	50	5.1
	10	50	5.2
2	1	50	8.2
	10	50	8.3
1	1	100	6.9
	10	100	7.0
2	1	100	9.8
	10	100	9.9

Expected average Specific Absorption Rate (SAR) for different values of rate of initializations per second *N*, rate of activations per second *n* and frequency of "Stimulation bursts" F.

The IEEE standard [24] specifies a maximum SAR of 20 W/kg at the extremities for persons in controlled environments (which is the scenario considered here as this corresponds to a medical system to be used under supervision). Therefore, according to the SAR estimations performed in (Table 1) in which the maximum obtained SAR (case in which we perform 2 initializations per second, with 10 activations per second and a frequency for “Stimulation bursts” of 100 Hz) would be 9.9 W/kg, the auxiliary current will safely comply with this restriction. These stimulation patterns seem adequate for many imaginable FES applications (frequency of stimulation pulses ≤ 100 Hz and less than 10 different activations per second). Actually, it needs to be taken into account that the IEEE standard indicates that the SAR has to be averaged for 6 minutes and it seems unlikely that any stimulation system will be continuously performing stimulation at 100 Hz for 6 minutes. Therefore, the safety margins may be actually considerably larger than what the values in Table 1 suggest.

For preventing unwanted electro-stimulation, the IEEE standard specifies that at 1 MHz the electric field magnitude at the extremities must be lower than 6.2 V_rms_/cm averaged for 0.2 s. With the dimensions considered for the numerical demonstration (inter-electrode separation distance of 3 cm), the peak electric field (6 V/cm) is already lower than this rms threshold. Therefore, the auxiliary current will comply with this restriction for any combination of *N*, *n* and F.

## *In Vivo* Demonstration: Materials and Methods

After electrical validation, the circuit prototypes for the microstimulators were tested in an *in vivo* model consisting of an anesthetized rabbit.

### Animal handling

The animal procedure was approved by the Ethical Committee for Animal Research of the Barcelona Biomedical Research Park (CEEA—PRBB), application number: JMC 14–1606. One New Zealand White male rabbit weighting 4 kg was employed in this study.

For sedation and initial anesthesia Dexmedetomidine (15 mg/kg), Butorfanol (150 μg/kg) and Ketamine (0.4 mg/kg) were intramuscularly administered. Then, prior to probe implantation and stimulation assays (later described), the left hindlimb of the animal was shaved, from the head of the femur to the mid tarsus. During implantation and stimulation assays, anesthesia was induced by delivering Sevofluorane using an oxygen mask, Ringer’s lactate was administered intravenously, a heating pad was employed, and the animal was constantly monitored with a capnograph and pulse oximeter.

### Materials and system configuration

In order to test the circuit prototypes *in vivo*, the motor point (muscle region of maximum electrical excitability) of the tibialis anterior (TA) and gastrocnemius (GA) muscles were located. The strategy is similar to that used in the BION implants [13]. At first, a 14 G intravenous catheter (Angiocath by Becton, Dickinson and Co.) was longitudinally introduced from a location close to the hock up to the proximal end of the TA muscle. The stainless steel introducer needle of the catheter was used as an exploration electrode, and an Ag/AgCl gel electrode (model 2228 by 3M Co.) located on the thigh of the animal was used as the return electrode. A custom made generator was used to generate conventional electrical stimulation with 2 to 5 V bipolar square pulses of 200 μs + 200 μs (cathodic first, no interphase dwell time) at 50 Hz. If the movement was considered not strong enough or did not match the expected dorsiflexion joint movement, the catheter was repositioned by pulling out or pushing in the catheter a few millimeters. Once the adequate motor point was located, the introducer needle was withdrawn. The same process was performed in order to locate the motor point of the GA muscle. Afterwards, two custom-made bipolar probes were inserted in these catheters assuring that the distal tip (where the stimulation electrode is located) was placed at the tip of the catheter. Finally, the catheters were pulled out and the probes were fixed to the skin using a hypoallergenic fabric bandage to avoid their movement. Each one of these two probes was then connected to one of the circuit prototypes for the microstimulators described in the Architecture of the Prototypes section.

**Fig 4:**
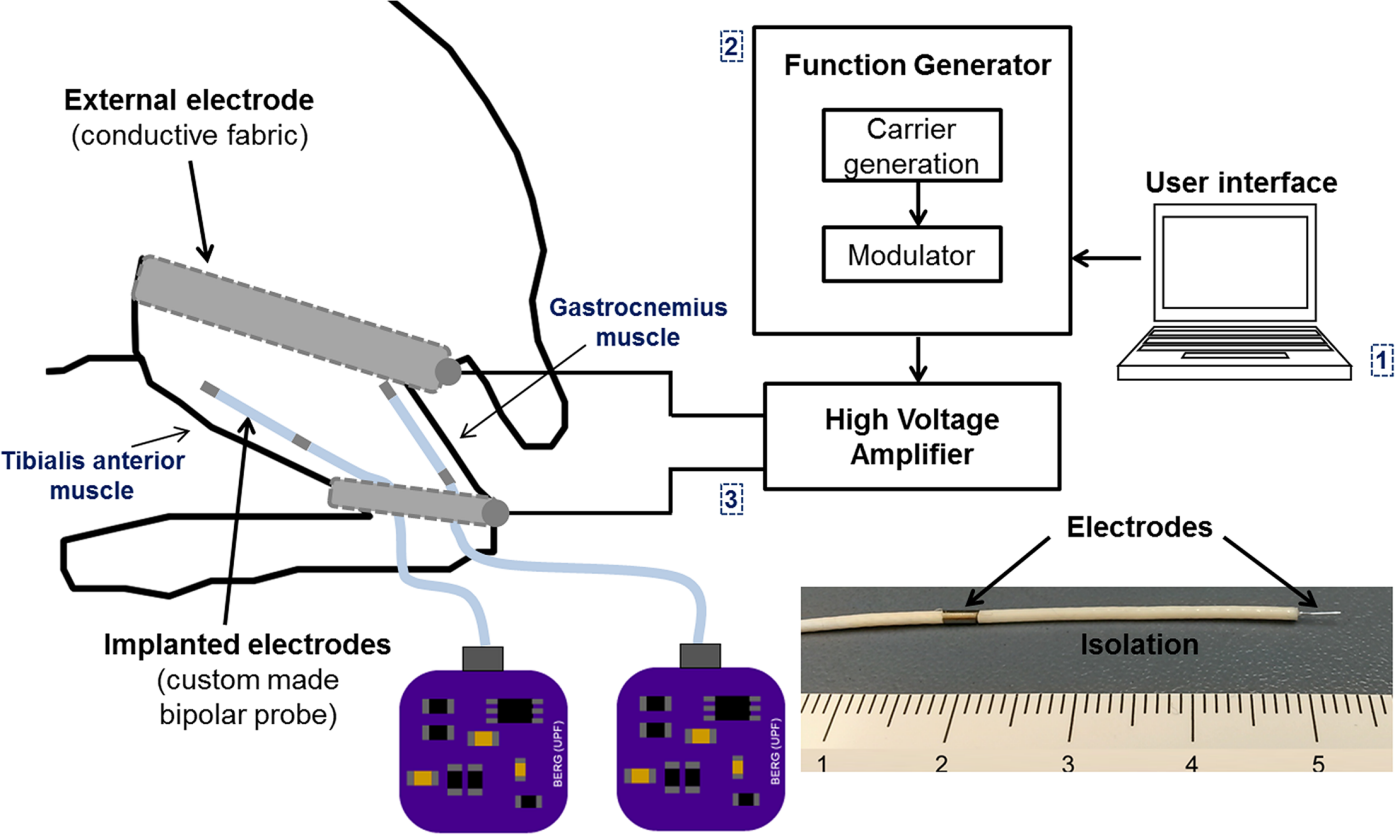
*In vivo* setup. It includes the external system (PC, function generator, high voltage amplifier and textile electrodes) and two prototypes connected to two bipolar electrode probes implanted in the tibialis anterior (TA) and the gastrocnemius (GA) muscles. Using this setup it is possible to independently perform electrical stimulation of either the TA muscle or the GA muscle of the rabbit in response to the commands of the experimenter.

The bipolar probes consist of a 1.17 mm diameter coaxial cable (Filotex ET087059 by Nexans S.A.) whose core conductor (silver plated copper covered steel wire of 0.17 mm diameter) is exposed for 3 mm at its distal tip. A 3 mm wide stainless steel ring of 1.3 mm in diameter has been placed in contact with the shield conductor at a distance of 3 cm from the tip. This forms a probe that consists of two cylindrical electrodes at a distance of 3 cm on a flexible shaft (Fig 4). The proximal tip of the coaxial cable (~ 50 cm) is soldered to a bipolar jack connector that can be plugged into the circuit prototypes. The distal electrode (at the tip) acts as the stimulation electrode whereas the proximal one, which is thicker, acts as the return electrode.

The external system consisted of three main concatenated parts: 1- a computer that generated the modulating signal in response to the commands and specifications indicated by the user on a graphic interface, 2- a carrier generator and modulator, and 3- a high voltage amplifier (Fig 4). The first part was implemented as a LabVIEW (National Instruments Corp.) virtual instrument running in a PC. It encoded the information defined by the user and generated a Manchester coded data stream that was sent to a modulator via a data acquisition (DAQ) board (NI-USB6216 by National Instruments Corp.). Then, this signal was used to modulate a 1 MHz sinusoidal voltage signal (carrier) using a function generator (AFG3022 by Tektronix, Inc.). The third part consisted of a high voltage amplifier (WMA 300 by Falco Systems).

**Fig 5:**
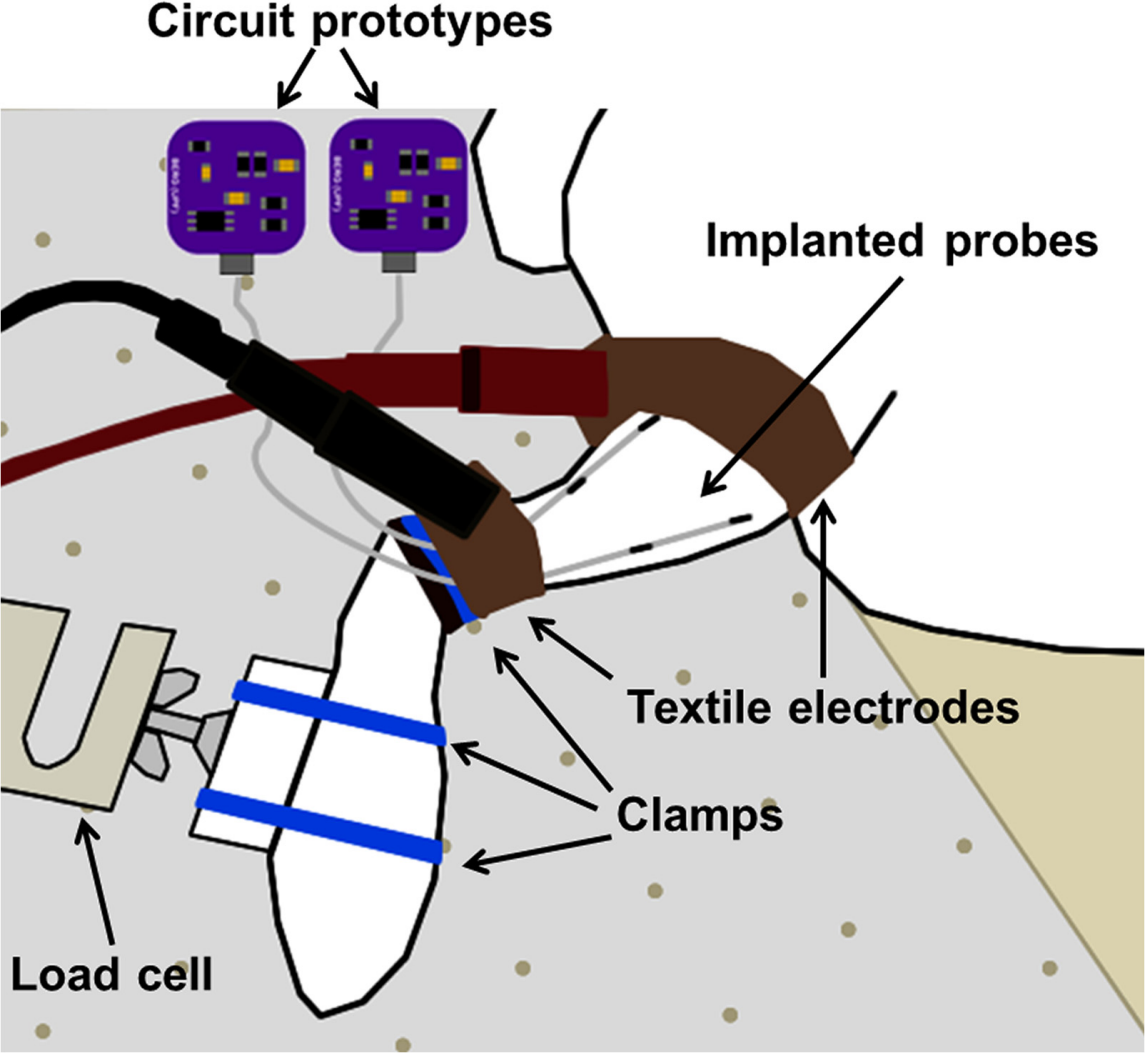
Force acquisition setup. The ankle of the animal is fixed on a horizontal surface and the foot is fixed to a load cell. Two textile electrodes strapped around the limb are connected to the high voltage amplifier in order to conductively supply high frequency current to the tissues.

The generated signal was delivered across a pair of 3 cm wide band electrodes made from silver-based stretchable conductive fabric (MedTex P-180 by Statex) strapped around the rabbit’s hindlimb where the bipolar probes were implanted (Fig 4 and Fig 5). The signal amplitude was relatively high (in the order of 50 V_peak_) and was adjusted so that the ratio between it and the average separation between the external electrodes was approximately 6 V/cm.

The low frequency components of the electric current flowing through a circuit prototype were obtained by recording with an oscilloscope (TPS2014 by Tektronix, Inc.) the voltage drop across the parallel combination of a 10 Ω resistor and a 2.2 μF capacitor (low-pass filter, cutoff frequency = 7.2 kHz) in series with the bipolar probe.

Isometric plantarflexion and dorsiflexion forces were recorded using a load cell (STC-10kgAL-S by Vishay Precision Group, Inc.) mounted on a custom-made acrylic board (Fig 5). The animal was positioned sideways. Its ankle was fixed to the board with an atraumatic padded clamp and its hind foot was tied to the load cell also with clamps. This setup is inspired by the torque measurement setup developed by Riso et al. in [29]. A LabVIEW virtual instrument recorded the load cell signal at a rate of 10 kHz by means of a DAQ board (NI USB-6211, by National Instruments Corp.) through a custom developed signal conditioning electronics which included a first order low-pass filter with a cutoff frequency of 500 Hz.

## Results

**Fig 6:**
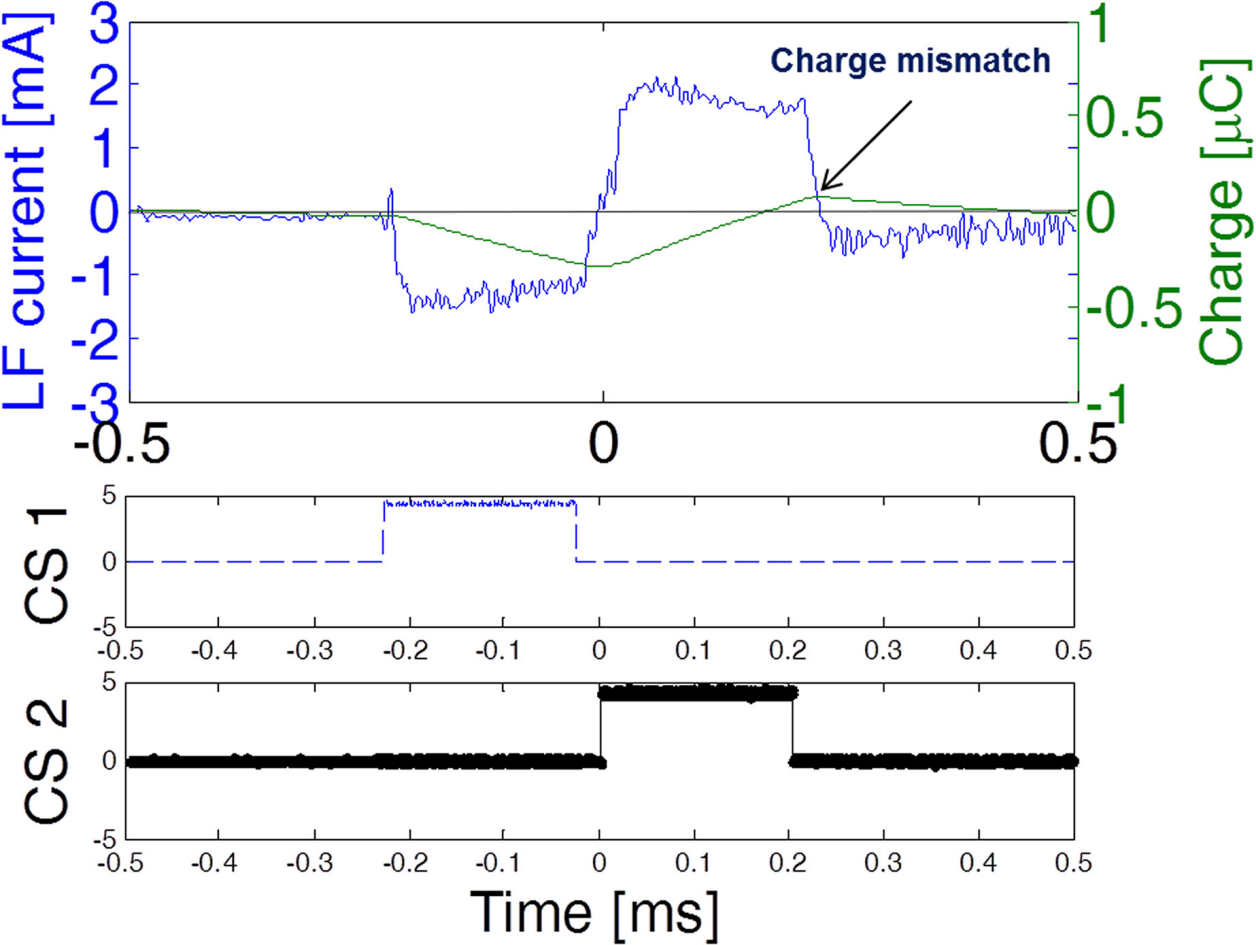
Low frequency current applied *in vivo* by a circuit prototype. Cathodic (negative) current was generated when control signal CS1 was active whereas anodic (positive) current was generated when control signal CS2 was active. A slight charge mismatch was present at the end of the biphasic pulse which was later passively balanced by the dc-blocking capacitor.

Fig 6 shows a stimulation biphasic pulse as recorded *in vivo* using the low-pass filter described in the Materials and Methods subsection, and its calculated charge injection. It can be observed that the circuit was able to apply pulses with an amplitude of 2 mA. The control signals (enable signals) generated by the microcontroller are also depicted for reference. A slight charge mismatch was present at the end of the biphasic pulse but it was later passively balanced by the dc-blocking capacitor.

**Fig 7:**
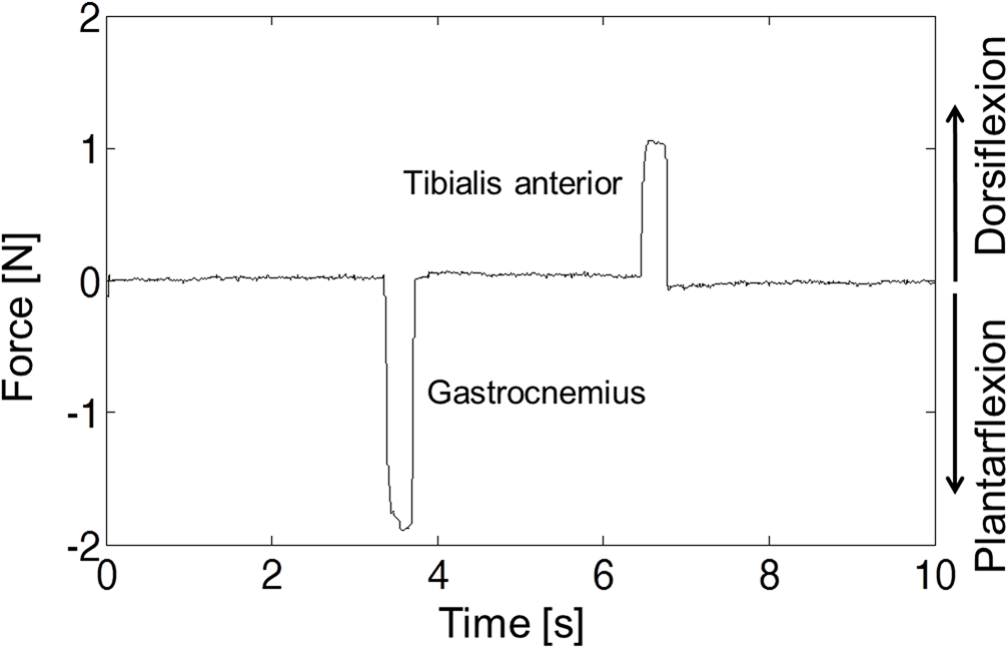
Addressability of the microstimulators. Force exerted on the load cell when the gastrocnemius and the tibialis anterior muscles were electrically stimulated by the addressable stimulators. This generated controlled plantarflexion and dorsiflexion joint movements.

We were able to induce either plantarflexion or dorsiflexion forces at will by clicking one or another button on the virtual instrument that governs the modulation of the HF signal. Fig 7 shows a trial example: first the circuit prototype connected to the bipolar probe implanted in the GA muscle was addressed and this generated a 1.9 N plantarflexion force on the load cell (F = 100 Hz, 30 bursts). Three seconds later, the second circuit prototype was addressed and this caused stimulation of the TA muscle with a 1.1 N dorsiflexion force. No force was exerted by the rabbit before, in-between or after triggered stimulations. This confirms that the circuits were capable of controlling the LF current for stimulation, and that the system was able to address each circuit at a time.

**Fig 8:**
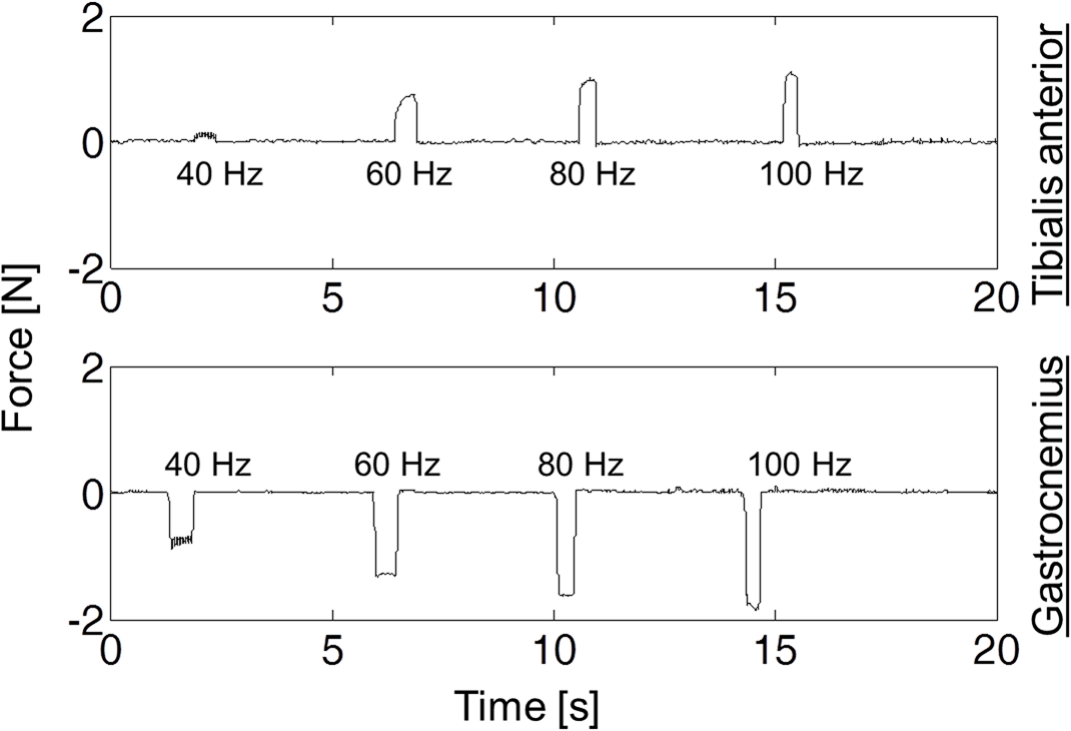
Force modulation capabilities. Force exerted on the load cell by independently stimulating the tibialis anterior and the gastrocnemius muscles. The magnitude of the force was modulated by varying the frequency of the stimulation bursts.

Fig 8 shows two trials in which the frequency of the stimulation bursts (F) was increased from 40 Hz to 100 Hz while the features of the biphasic pulse were kept constant. It can be observed that the force exerted by the foot on the load cell increased with the frequency of the bursts. That is, force modulation was not only possible by varying the amplitude of the pulses or their duration (results not shown here) but also by varying the repetition rate of the stimulation bursts. This is a common observation in neuromuscular electrical stimulation when regular low frequency pulses are employed [30]. Therefore, this seems to indicate that the outcome of the currents generated by the devices (rectified HF current bursts) was equivalent to that of regular current pulses.

## Discussion

With the goal of achieving miniaturization and deployment simplicity, we propose an electrical stimulation method in which implanted microdevices electrically rectify innocuous HF current bursts flowing through the tissues so that LF currents capable of stimulation are generated locally through the microdevice. In their simplest form, those microstimulators can consist of just a diode and two peripheral electrodes (referred to as “muscle electrodes”). This concept, which we independently envisioned in “Remote Electrical Stimulation by Means of Implanted Rectifiers” [16], was in fact first proposed in the 60s by at least two independent research teams in two infrequently cited studies [31,32]. However, to the best of our knowledge, neither advanced rectifiers with communication capabilities nor simple rectifiers capable of blocking DC current have been proposed until now. In historical perspective it is easy to understand such neglect: at the time microelectronics was at its infancy and, therefore, the coils and batteries were not the miniaturization bottleneck but the electronics.

Communications, and in particular addressability, are a requirement if multiple targets must be stimulated independently in a controlled manner. This would be the case of most FES systems in which it is required to act on multiple motor points. In comparison to stimulation systems based on central units, a wireless network of addressable microstimulators may offer a much higher number of independent stimulation sites. Then, it will be possible to independently stimulate small groups of motor units in a muscle, achieving finer recruitment of muscle fibers.

Here we have demonstrated that neuroprosthetic systems composed of addressable microstimulators that are based on the proposed electrical stimulation method are feasible. In particular we have shown that the microstimulators are able to perform independent stimulation of agonist and antagonist muscles in the rabbit’s hindlimb. Moreover we have observed that the stimulation behavior seems to be equivalent to that that would be obtained using regular LF pulses. This was the case of the custom made generator used to locate the motor points of the TA and GA muscles. More complex stimulation patterns would be needed to improve muscular stimulation. This implies activating the implants sequentially, synchronically, or during overlapping periods of time. The electronic architecture proposed in here may be able to include these stimulation capabilities; only significant differences would appear in the amplitude modulated HF signal and in the control unit’s algorithm. We plan to carry out additional research on this topic.

The above demonstration has been achieved by applying an amplitude modulated HF (1 MHz) voltage across two textile band electrodes strapped around the hindlimb. We also numerically demonstrated that this resulting current flowing through the tissues (which can be understood as an auxiliary current) complies with the relevant IEEE safety standard. This innocuous current conductively powers the circuit prototypes, conveys information (address of the circuit to be activated) and it becomes the stimulation current when it is rectified through the circuits. It is worth noting that the demonstration presented here focused on technical aspects, proving that the neuroprosthetic system includes digital addressability and can perform electrical stimulation.

The stimulation systems described up to this point are open-loop systems: preprogrammed stimulation patterns are executed on command. By implementing uplink communications (from the microstimulators to the external system), it is possible to conceive closed-loop systems in which the stimulation signals are modulated in response to measurements performed with the implants. For instance, the level of muscle contraction (e.g. by measuring the myoelectric activity or the pressure) or the joint angles (e.g. by performing magnetic field goniometry) are data that could be employed for performing more natural movements [13]. These are topics on which we intend to carry out further research.

Table 2 compares three different types of FES configurations (i.e. surface, percutaneous and implantable) in terms of: 1. surgical simplicity, 2. selectivity, 3. safety, 4. usability (i.e. ease to don and doff) and 5. ability to perform stimulation in muscle. Superficial and percutaneous configurations are not appropriate for fine muscle recruitment in long-term applications. This is explained as the former lacks selectivity, and the latter presents low safety and usability levels [9]. Implantable central units are able to accomplish high selectivity [33]. Yet this system presents surgical complexity and low safety levels due to leads that run through the tissues [13]. To overcome this, distributed implantable systems (DIS) make use of multiple wireless microstimulators. These systems offer high selectivity as they are located near motor points (e.g. inductive coupling, batteries or rectification of epidermically applied currents), or near the neural tissue (e.g. infrared).

**Table 2:** Comparison of Configurations for FES Systems.

					Usability	Ability to
System configuration		Surgical simplicity	Selectivity	Safety	(ease to don and doff)	perform stimulation in muscle
**Superficial**		+++++	+	++++	++	+++++
(external pulse generator connected to skin electrodes)		(no surgery needed)	(difficulty for isolated contractions and deep muscle activation; may activate pain fibers [9])	(skin electrodes are driven by external system)	(every use implies don and doff; difficult to position for adequate stimulation [9])	
**Percutaneous**		+	+++	+	+	+++++
(external pulse generator; the skin is pierced by the leads and the electrodes are anchored near the motor points)		(skin piercing and electrode anchoring)	(electrodes can displace due to traction forces in the leads)	(possible infections due to skin piercing)	(used on research and clinical diagnosis [9])	
**Implantable central unit**		++	+++++	++	++++	+++++
(the implantable pulse generator is connected to electrodes using leads. The generator is powered using batteries [33])		(implantation of generator and leads that run through tissues; electrode anchoring)	(electrodes are placed near motor points)	(possible infections due to leads that run through tissues [13])	(only one anatomical point is required for programming using radiofrequency. This is also used for battery recharging)	
	**Powering Method**					
	**Inductive Coupling**	++++	+++++	+++	++	+++++
	(ø 2 mm) [35]	(deployment using thick catheter)	(microstimulators are placed near motor points)	(possible foreign body response)	(external and implanted coils have to be coupled)	
**Distributed implantable system**	**Battery**	++++	+++++	+++	+++	+++++
	(ø 3.15 mm) [13]	(deployment using thick catheter)	(microstimulators are placed near motor points)	(possible foreign body response)	(radiofrequency used for battery recharge of each microstimulator)	
(the pulse generator and electrodes are housed inside an implantable package. Multiple wireless stimulators are placed in a small anatomical area)	**Infrared**	++	+++++	++	++++	+
	(optical fibers used to power photodiode microstimulators in CNS.) (ø ≤ 0.2 mm) [34]	(implantation in CNS (e.g. spine); optical fibers must be anchored close to microstimulators)	(microstimulators are placed in neural tissue for intraspinal stimulation)	(possible infections in optical fibers)	(only one radiofrequency link is used for powering and programming implantable central unit)	(do not deliver enough current for neuromuscular stimulation; only for intraspinal stimulation (≤ 120 μA))
	**Rectification of epidermically applied currents**	+++++	+++++	+++	+++	+++++
	(ø ≤ 0.5 mm)[method demonstrated in this study]	(deployment using **thin** catheter)	(microstimulators are placed near motor points)	(possible foreign body response)	(external electrodes added to clothes; portable external system [26])	
Meaning of qualifiers: + stands for poor, whereas +++++ stands for excellent.						

Superficial, percutaneous and implantable systems are compared in terms of surgical simplicity, selectivity, safety, usability and their ability to perform stimulation in muscle. The implantable systems are divided depending if they use central units, or they make up a network of distributed wireless microstimulators.

Infrared DIS offer advantages in terms of selectivity and usability. However, this system is not able to drive enough current for muscular stimulation. It is used for intraspinal stimulation [34], as the microdevices drive less than 120 μA. In addition, as the system is implanted in the central nervous system (CNS), it presents low surgical simplicity and safety levels.

The DISs that are implanted near motor points offer high selectivity and are able to drive enough current to perform stimulation in muscles. The microstimulators based on the electrical stimulation method proposed here present high levels of surgical simplicity and usability. As we can accomplish thinner implants, the deployment of the wireless microstimulators is much simpler than that offered by implants based on inductive coupling or batteries.

In terms of usability, we have been able to demonstrate that the external system can be portable [26]. In addition, skin electrodes made of conductive fabric can be easily added to clothes. This imposes an advantage over systems powered by inductive coupling: whereas the first method depends on the skin electrodes, in inductive coupling the implants will only power up and stimulate when the external and the implanted coils are coupled.

The results demonstrate for the first time that wireless microstimulators powered by rectification of epidermically applied currents are able to stimulate excitable tissue in a vertebrate model, accomplishing addressability and force modulation.

## Conclusions

It has been demonstrated that a neuroprosthetic system composed of addressable microstimulators based on the electronic rectification of epidermically applied currents is feasible. The electrical stimulation method proposed is safe, realizable and the prototypes can perform controlled electrical stimulation of muscles in a vertebrate model. The system is able to passively balance the charge injected during stimulation and modulate force by varying the frequency of the stimulation bursts.

## Supporting Information

S1 Video Supplementary video of *in vivo* demonstration. The supplementary video shows the movement of the rabbit’s hindlimb when the gastrocnemius and tibialis anterior muscles are electrically stimulated. In this experimental setup, the limb freely moves over a horizontal surface. The video first describes the setup and the connections made for the external system and the circuit prototypes, and then shows two sequences of stimulation.
(MP4)
